# Automated Knowledge-Based Intensity-Modulated Proton Planning: An International Multicenter Benchmarking Study

**DOI:** 10.3390/cancers10110420

**Published:** 2018-11-02

**Authors:** Alexander R. Delaney, Lei Dong, Anthony Mascia, Wei Zou, Yongbin Zhang, Lingshu Yin, Sara Rosas, Jan Hrbacek, Antony J. Lomax, Ben J. Slotman, Max Dahele, Wilko F. A. R. Verbakel

**Affiliations:** 1 Cancer Center Amsterdam, Department of Radiation Oncology, VU University Medical Center, De Boelelaan 1117, 1081 HV Amsterdam, The Netherlands; bj.slotman@vumc.nl (B.J.S.); m.dahele@vumc.nl (M.D.); w.verbakel@vumc.nl (W.F.A.R.V.); 2 Department of Radiation Oncology, University of Pennsylvania, Philadelphia, PA 19104, USA; Lei.Dong@uphs.upenn.edu (L.D.); Wei.Zou@uphs.upenn.edu (W.Z.); Lingshu.Yin@uphs.upenn.edu (L.Y.); 3 Department of Radiation Oncology, University of Cincinnati Medical Center, 234 Goodman Street, Cincinnati, OH 45219, USA; Anthony.Mascia@cchmc.org (A.M.); Yongbin.Zhang@UCHealth.com (Y.Z.); 4 Paul Scherrer Institute, Center for Proton Radiotherapy, 5232 Villigen, Switzerland; Sara.Rosas@psi.ch (S.R.); jan.hrbacek@psi.ch (J.H.); tony.lomax@psi.ch (A.J.L.)

**Keywords:** proton therapy, IMPT, head and neck cancer, knowledge-based planning, model-based planning, automated

## Abstract

**Background:** Radiotherapy treatment planning is increasingly automated and knowledge-based planning has been shown to match and sometimes improve upon manual clinical plans, with increased consistency and efficiency. In this study, we benchmarked a novel prototype knowledge-based intensity-modulated proton therapy (IMPT) planning solution, against three international proton centers. **Methods:** A model library was constructed, comprising 50 head and neck cancer (HNC) manual IMPT plans from a single center. Three external-centers each provided seven manual benchmark IMPT plans. A knowledge-based plan (KBP) using a standard beam arrangement for each patient was compared with the benchmark plan on the basis of planning target volume (PTV) coverage and homogeneity and mean organ-at-risk (OAR) dose. **Results:** PTV coverage and homogeneity of KBPs and benchmark plans were comparable. KBP mean OAR dose was lower in 32/54, 45/48 and 38/53 OARs from center-A, -B and -C, with 23/32, 38/45 and 23/38 being >2 Gy improvements, respectively. In isolated cases the standard beam arrangement or an OAR not being included in the model or being contoured differently, led to higher individual KBP OAR doses. Generating a KBP typically required <10 min. **Conclusions:** A knowledge-based IMPT planning solution using a single-center model could efficiently generate plans of comparable quality to manual HNC IMPT plans from centers with differing planning aims. Occasional higher KBP OAR doses highlight the need for beam angle optimization and manual review of KBPs. The solution furthermore demonstrated the potential for robust optimization.

## 1. Introduction

The number of proton centers is increasing [1]. However, treatment planning studies for established photon modalities such as intensity-modulated radiation therapy (IMRT) and volumetric-modulated arc therapy (VMAT) indicate substantial intra- and inter-institutional variation [2]. This will presumably also be the case for intensity-modulated proton therapy (IMPT) since IMPT treatment planning is complex and involves many steps, including: determining beam arrangements, using appropriate optimization objectives and deciding whether to use of a range shifter or bolus. While certain centers are using robust optimization, which adds further complexity, the majority of proton therapy treatments, to-date, have been delivered without this [3,4,5,6]; and although online adaptive proton therapy has been proposed to deal with daily anatomical changes, this requires faster planning solutions to be practical.

A desire to reduce variation, and produce consistently “good” plans, has largely spurred the development of automated treatment planning for photon therapy [7,8,9,10]. One such approach is knowledge-based planning, an example of which is RapidPlan^TM^ (Varian Medical Systems, Palo Alto, CA). RapidPlan utilizes the geometries and associated dosimetry of previously created treatment plans to construct a model which can be then used to predict a range of achievable organ at risk (OAR) dose-volume histograms (DVHs) for future patients. Provided the library of plans used to derive the model is of a “high enough” quality, improvements in photon treatment plan quality and reductions in planning time and variability are attainable. Knowledge-based planning has also demonstrated a role in patient-specific plan quality assurance (QA) [11,12,13,14]. 

We previously illustrated the principle of using the photon-RapidPlan solution to automatically create IMPT treatment plans [15]. Since then, we have collaborated on the development of a prototype knowledge-based IMPT (proton-specific) planning solution, RapidPlanPT, in which the physical characteristics of proton beams have been appropriately modeled. In this novel study, we benchmark automated knowledge-based plans (KBPs) from this prototype solution against manual head and neck cancer (HNC) IMPT plans from 3 international protons centers. By using a model based on plans from a single center we also evaluate the versatility of such an approach.

## 2. Results

**Figure 1:**
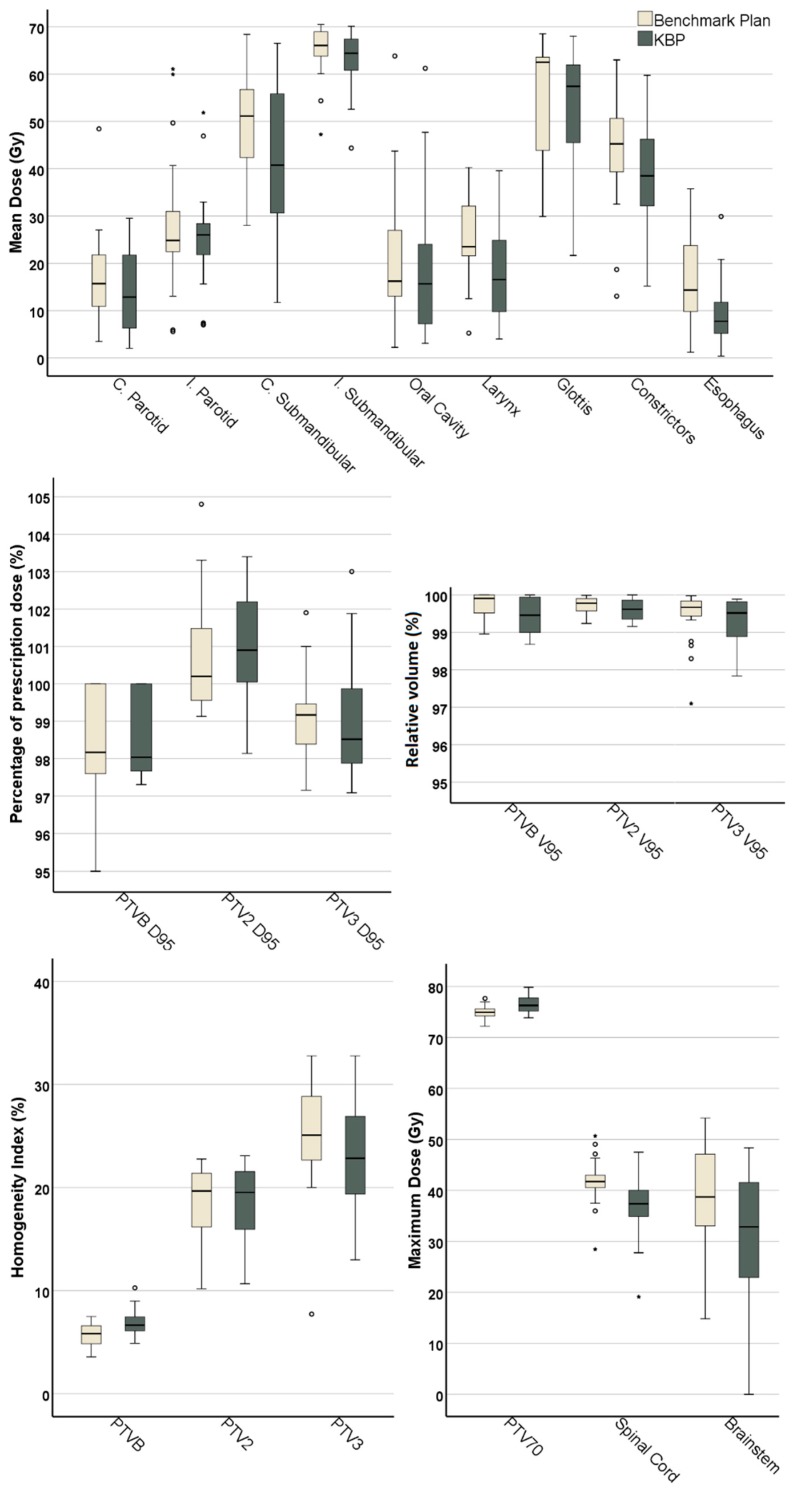
Box-whisker plots for all 21 patients from external centers. Dark-line in the middle of box denotes the median while the top/bottom of the box indicates 75th/25th percentile. A structure denoted with “*” indicates a statistically significant difference between benchmark plans and knowledge-based plans (KBPs) for said structure.

**Figure 2:**
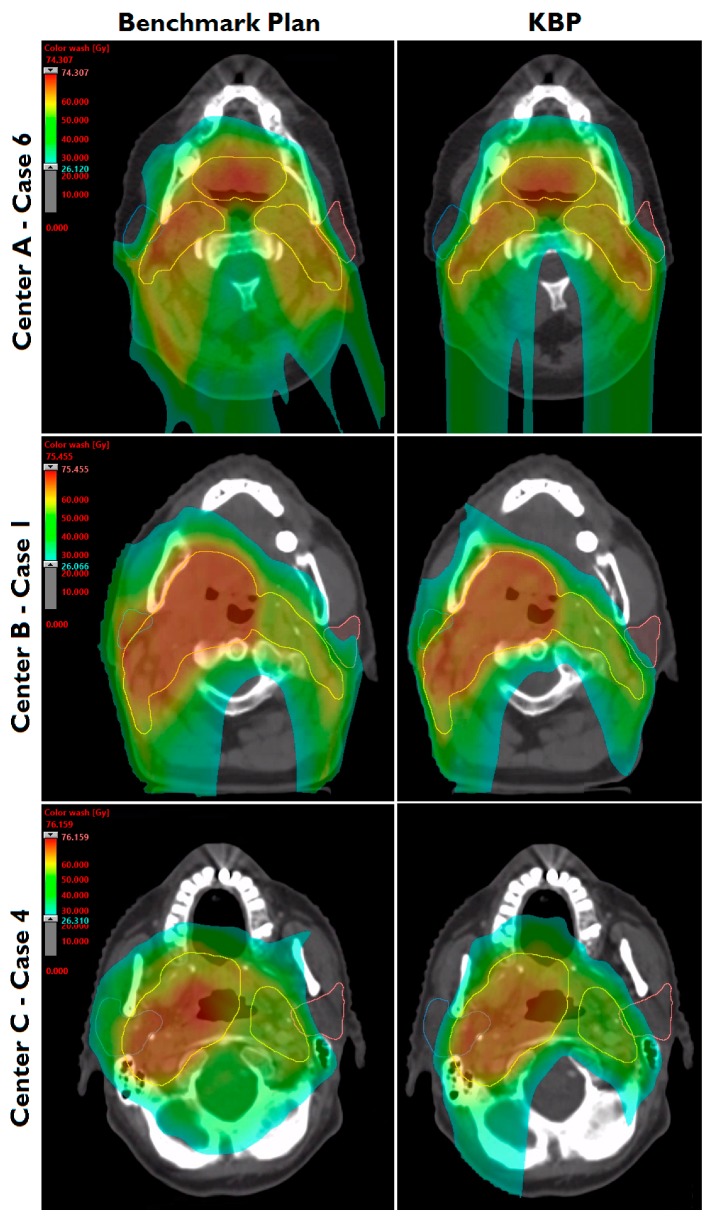
Dose distributions of benchmark plans and knowledge-based plans (KBPs) for one evaluation patient from each external center. Parotid glands (blue, pink) and the composite planning target volume (yellow) are shown.

**Figure 3:**
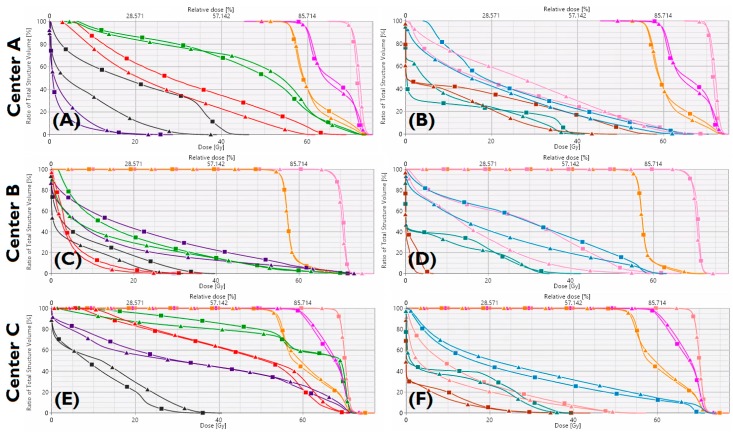
Benchmark plan (■) and KBP (▲) dose-volume histograms for one patient from Center-A (**A**,**B**), Center-B (**C**,**D**), Center-C (**E**,**F**). Targets: PTV_B_(Pink), PTV_E1_(magenta), PTV_E2_(orange). Organs-at-risk: brainstem (dark-grey), oral cavity (purple), constrictors (green), larynx (red), esophagus (brown), left parotid (pink), right parotid (blue), spinal cord (petroleum-blue).

Summarized results for the 21 patients from all external centers are presented in Figure 1. Generally, target coverage and homogeneity were similar between KBPs and benchmark plans. Aside from the oral cavity and ipsilateral submandibular gland, KBP OAR mean dose was >2 Gy lower, on average, than in benchmark plans, with statistically significant differences in a number of OARs including the contralateral submandibular gland (8.6 Gy) and larynx (8 Gy). Figure 2 and Figure 3 show instances of similarities and differences in dose distributions and DVHs between benchmark and KBPs. Generation of DVH-predictions, subsequent optimization and dose calculation of KBPs required 8.3 min (*n* = 6 plans). Table 1 shows the dosimetry for the seven benchmark plans and KBPs from each external center.

**Figure 4:**
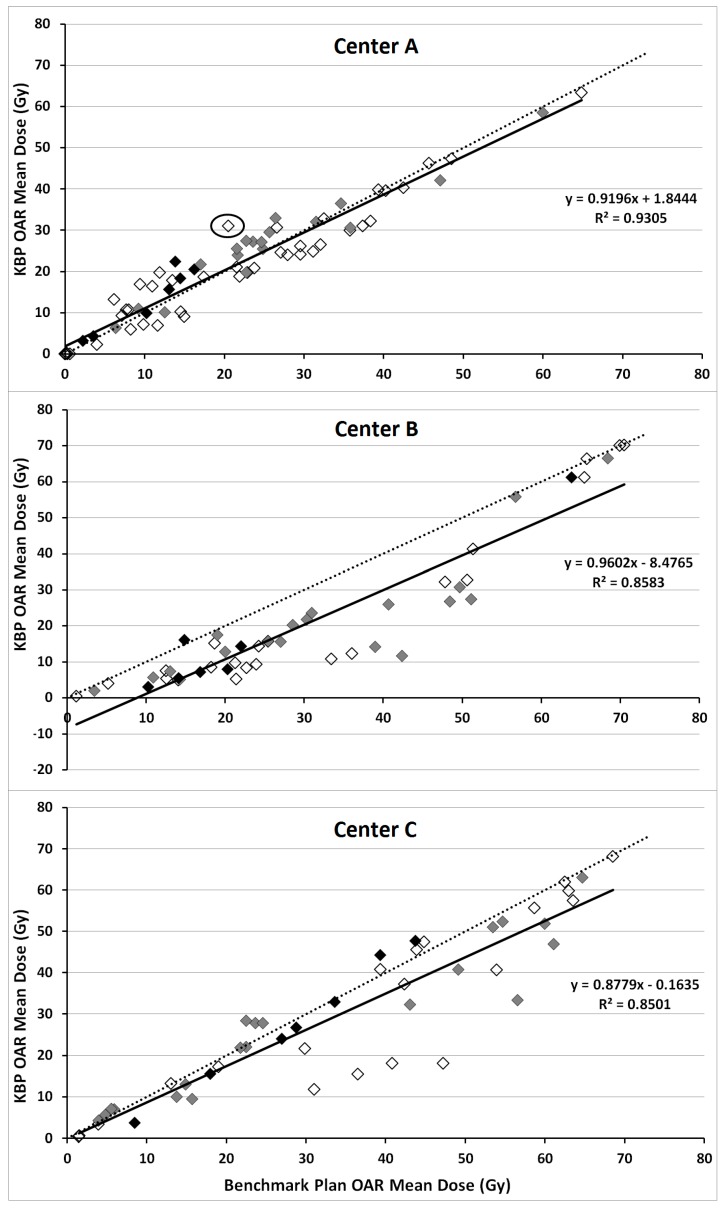
Correlation between mean dose of the salivary glands (grey), oral cavity (black) and all other OARs listed under “mean dose” from Table 2 (white) in knowledge-based plans (KBPs) (vertical-axis) and respective benchmark plans (horizontal-axis). Dotted line indicates line through the origin with slope of 1 while solid line represents linear fit through data points.

For center-A, all but one KBP met the aims for PTV_B_ Dmax of ≤110% (patient 1 had a Dmax of 111% delivered to a volume of 0.1 cm^3^, however center-A accepts >110% in 0.03 cm^3^). While benchmark plans had, on average, lower parotid gland and oral cavity mean dose, KBPs had lower submandibular gland, esophagus and larynx mean dose. A “continue optimization” was performed to improve PTV homogeneity in four cases while in two cases it was performed for non-modeled OARs not meeting the criteria in Table 2 (assigned the maximum accepted dose as an objective). Figure 4 illustrates the similarity between the mean dose values of OARs (included in Table 2) in benchmark plans and KBPs, with the R^2^ value, 0.91, and slope, being close to 1. One OAR, for which the KBP resulted in considerably higher dose than the respective benchmark plan, is circled in Figure 4. This was a left ear with a KBP mean dose of 31 Gy (aim <30 Gy). The largest increase in mean dose to a salivary gland, when using KBPs over benchmark plans for center-A, concerned an ipsilateral parotid gland (6.6 Gy). This increase was due to the standard, Y-shaped, beam arrangement used in the KBP compared with a five-field technique, including two oblique dorsal fields, in the benchmark plan.

All center-B KBPs met PTV_B_ planning-aims despite a small, but statistically significant, decrease in PTV_B_ V95% (Table 1). On average, KBP PTV_E1_/PTV_E2_ V95% was 0.7/0.8% lower than in the benchmark plan. KBPs improved all OAR metrics in Table 1, with a ≥8.2 Gy statistically significant reduction in mean dose to the parotid glands. The largest, statistically significant, improvements in sparing, on average, were in the contralateral submandibular gland (16.3 Gy) and larynx (12.6 Gy). A “continue optimization” was performed to improve PTV homogeneity in all cases however it was not required in any case to improve dosimetry for OARs excluded from the model. For instance, in three patients PTV_E2_ did not meet the planning-aims of V95% ≥99% after one round of optimization. This was resolved by, after optimization, converting any cold spots in PTV_E2_ into contours and running the “continue optimization”, including additional objectives for the cold spots, ensuring planning-aims were met. 

All center-C KBPs met PTV coverage-aims (D95% ≥95%), with minimal differences in PTV coverage and homogeneity relative to benchmark plans. Averaged over seven patients, KBPs improved all OAR sparing (Table 1); noticeable improvements occurred in the contralateral submandibular gland (8.1 Gy) and constrictor muscles (10.6 Gy). There was good correspondence of mean dose to the oral cavity between KBPs and benchmark plans (Figure 4). A “continue optimization” was performed to improve PTV homogeneity in all cases, two of which it was also used to improve dosimetry for OARs excluded from the model. However, for these two instances, in contrast to the benchmark plans, the KBP did not meet planning-aims and resulted in inferior sparing: (1) the V_54 Gy_ for the left brachial plexus of patient six was 1.1 cm^3^ (aim ≤0.05 cm^3^) and (2) the V_52 Gy_ for the right brachial plexus of patient seven was 0.6 cm^3^ (aim ≤0.05 cm^3^) when using the KBP.

## 3. Discussion

This study, aimed at providing proof-of-principle, demonstrated that a knowledge-based planning solution, using a model library comprising plans from a single center with a standard planning technique, could create good quality HNC IMPT plans when benchmarked against plans from three experienced proton centers. The slightly different planning-aims of each center demonstrated the versatility of this automated approach. Creation of KBPs was efficient, requiring <10 min for generating predictions, subsequent optimization and dose calculation. Occasional higher OAR doses in KBPs highlights the need for manual review of KBPs and an understanding of the limitations of such a knowledge-based approach using fixed beam orientations and clinic-specific planning-aims. Knowing how “good” the plans are in a model library remains a limitation with approaches like that used in RapidPlan/RapidPlanPT. This is one reason why plans from multiple experienced proton centers have been used to benchmark the KBPs in this study. The fact that KBPs were at least comparable to or better than the benchmark plans, indicates that model library plans were of a reasonable quality. 

For OARs, common to both the model and benchmark plans, KBPs provided comparable or improved sparing over respective benchmark plans. However, certain OARs/PTVs had an inferior dose distribution in KBPs. In some cases, this concerned OARs not included in the model, such as the ear of center-A and brachial plexus of center-C. For such OARs the maximum accepted dose in the planning aim was used as an objective in the KBP. However, because it was not attempted to reduce dose below this, and because in some cases the optimization weighting may have been too low, dose may have been higher than necessary in the KBP. This reflects a limitation when using a clinic-specific model for other centers: not all OARs may be the same. Furthermore, center-A oral cavity delineation was inconsistent, due to inter-physician differences, and the higher oral cavity mean dose (13.5 Gy versus 10.5 Gy) for KBPs of center-A may be attributable to the delineation of the oral cavity in patients 1–4. These oral cavity structures were >200% larger than cases 5–7, resulting in on average 4.4 Gy higher doses in the KBPs compared with benchmark plans. Although, visually, these contours differed from those in the model, the software did not flag them as volumetric outliers. Patients 5–7 had, on average, only 1.1 Gy higher oral cavity dose in KBPs. While these increases in mean dose may not be substantial, users should nonetheless check that OAR delineation/geometry of a prospective patient is similar to that in the model.

Since modeling partly depends on the beam-arrangement of plans in the model library, it was decided that in order to obtain the most accurate predictions for prospective patients, KBPs should have the same beam-arrangement as those in the model-library. Therefore, this study shows the result of straight-forward, standard three-field automated IMPT optimization without beam angle optimization. This could be a limitation of the study and KBP plan quality may be further improved with patient-specific beam-arrangements (as in the benchmark plans). Future investigations should test the applicability of such a model for differing beam arrangements [16] and include differing beam-arrangements in the model library. Optimized beam angle selection in IMPT is under investigation [17,18] and could potentially be integrated into future IMPT planning solutions. Since all external centers used the same treatment planning system, the versatility of our approach, with respect to plans from multiple vendors, was not tested. Furthermore, external centers used a bolus/range shifter, and differing beam data [19,20] and target margins. While examining the effect of such parameters on the results was beyond the scope of this study, previous work has alluded to these issues. Langner et al. compared commissioning beam data for two separate proton treatment centers which obtained their beam data using independent dosimeters and found that spot profiles were very similar both intra-institutionally (between treatment rooms) and inter-institutionally [21]. Minimal outlier removal was performed in the current analysis and while previous work has indicated that outlier removal may not always be necessary for RapidPlan photon KBPs [22,23], this has not yet been tested for RapidPlanPT. Finally, we note that the software used in this study is still under refinement and improvements are possible. 

The KBPs in this study were non-robustly optimized, consistent with general clinical practice at the outset of this study [3,4,5,6]. The results are therefore relevant to contemporary practice. Whether RapidPlanPT can also be used for robust optimization requires further investigation. Since center-A began using robust optimization during the course of this study, and interest in robust optimization is increasing, we performed a preliminary test of the potential of using RapidPlanPT with a model based on non-robustly optimized plans to be used for robust optimization. Center-A provided clinical, robustly optimized plans for three new cases. Using the same model (comprising non-robustly optimized treatment plans), we created robustly optimized KBPs for these patients. All three KBPs met center-A’s robustness criteria and plan quality was largely comparable to that of the clinical plans (Supplementary Materials: Figures S1 and S2, Tables S1 and S2). These initial results are encouraging and merit investigation with a larger sample size. 

We previously showed the feasibility of using a photon-specific knowledge-based solution for IMPT [15]. The present study incorporates proton-specific software which appropriately characterizes proton behavior. Literature on automated proton planning solutions is relatively sparse and we are currently unaware of similar multi-institutional comparisons. However, Hall et al. used OAR-PTV geometry to predict achievable doses for OARs relevant to the treatment of skull-base tumors in order to facilitate comparison of proton and photon plans [24]. Bijman et al. looked at uncertainties in model-based patient selection for IMRT or IMPT, using automatically planned IMPT plans: the approach to automation differed from this study, in that it used a pre-defined wish-list of hard constraints and hierarchical OAR objectives (tackled in order of priority) [25]. Lomax and colleagues have developed a tool to automatically pre-calculate feasible planning solutions specifically for uveal melanomas [26]. 

## 4. Conclusions

This proof-of-principle study has shown that a knowledge-based approach to IMPT planning is feasible. Such a solution could aid both experienced and more recently established proton centers, with a number of potential applications including implementation of consistent, efficient treatment planning; fast (re)planning for adaptive IMPT; and QA of IMPT plans in the clinic and trial setting [27].

## 5. Materials and Methods 

### 5.1. Treatment Planning for Populating the Knowledge-Based Planning Model

A constant radiobiological effectiveness (RBE) of 1.1 was assumed for IMPT plans, where all doses reported in “Gy” are understood to represent Gy_RBE_ [28]. The 50 non-nasopharynx, locally advanced HNC IMPT plans in the RapidPlanPT model were coplanar and used a simultaneous integrated boost (SIB) technique to deliver 70/54.25 Gy to the boost/elective planning target volume (PTV_B_/PTV_E_) in 35 fractions. PTV margins were 4–5 mm [29]. A 5 mm transition-region facilitated dose fall-off between PTVs. Plans typically aimed to spare multiple salivary glands, swallowing muscles and the oral cavity [30], although certain OARs could be excluded due to the extent of overlap with PTVs. Maximum point dose objectives were used for spinal cord, brainstem and planning-at-risk volumes (isotropic 3 mm OAR expansion). The aim was to deliver 95% of prescribed dose (V95%) to ≥99%/98% of PTV_B_/PTV_E_ while limiting PTV volume receiving >107% of prescription dose. IMPT plans were created using the Varian Eclipse non-linear universal proton optimizer (NUPO) and proton convolution superposition algorithm (PCS) v13.7.14 (Varian Medical Systems, Palo Alto, CA, USA) with a 2.5 mm calculation grid. Plans were made with a standard three-field, multi-field optimization (MFO) technique, with gantry angles at 35°–55°, 180° and 305°–330°. Gantry variations in these three directions were determined by PTV geometry. A range shifter of 5.7 cm water equivalent thickness was used for proximal PTV irradiation [31]. Each field included proximal, distal and lateral target margins of 0.2 cm, 0.3 cm and 0.5 cm, respectively. A non-robust optimization was performed interactively by manually adjusting optimization objectives to maintain an approximately fixed diagonal distance to DVH-lines in the optimization-window. In certain cases, a subsequent optimization or “continue optimization” was performed to improve PTV dose homogeneity [32]. A basic refinement of the model was carried out in which regression, residual and DVH-plots, as well as statistical metrics provided by the planning software were used to remove obvious outliers with DVHs a considerable distance above the predicted curve [14,22]. The minimal refinement is reflected in the number of structures matched to each OAR in the model (average: 47, minimum: 37).

### 5.2. External Center Treatment Planning

Typical planning-aims for PTVs and OARs are shown in Table 2. Center-A defined dose-volume constraints on “OAR minus PTV” structures whilst center-B and -C used the entire OAR. For analysis, all dosimetric data is reported on the entire OAR structure. Up to two elective PTVs, which could differ in prescription dose per patient, were used. Therefore, we refer to the boost PTV as PTV_B_, the mid-dose elective PTV as PTV_E1_, and the low-dose elective PTV as PTV_E2_. This study intends to demonstrate the feasibility of knowledge-based planning for IMPT, regardless of the beam data used. All reporting is done on the PTVs provided by each center. Although differences in beam data and the choice of proximal/distal spot placement margins may have some effect on the dose distribution and amount of OAR sparing, such effects are expected to be small for state of the art clinical IMPT facilities, and the choice of optimization objectives is likely to have more impact on the OAR sparing. For all these reasons the KBP is considered relatively insensitive to the vendor-specific beamline or beam model.

#### 5.2.1. Center-A

IMPT plans utilized a SIB, delivering 70 Gy, 63/59.5 Gy and 56/54.3 Gy to PTV_B_, PTV_E1_ and PTV_E2_ in 35 fractions, respectively. PTV aims were such that dose to 95% of the volume (D95%) was ≥100% of the prescribed dose. IMPT plans were created using Varian Eclipse NUPO and PCS v13.7.15 with a 2.5 mm dose calculation grid. Plans utilized a three or five-field MFO technique, including a beam from the anterior direction to irradiate the neck and shoulder nodal regions and two oblique posterior-anterior beams utilized for the head, however, beam angles varied according to tumor location. A range-shifting bolus of 7.5 cm water equivalent thickness was used in all plans. Each field included proximal, distal and lateral target margins of typically 0.5 cm, 0.5 cm and 0.3–0.5 cm, respectively.

#### 5.2.2. Center-B

IMPT plans utilized a SIB, delivering 70 Gy, 63 Gy and 56 Gy to PTV_B_, PTV_E1_ and PTV_E2_ in 35 fractions, respectively. The aim was that all PTVs had a V95% ≥ 99%. IMPT plans were created using Varian Eclipse NUPO and PCS v13.7.15 with a 2.5 mm dose calculation grid. Plans utilized a three-field MFO with typical gantry angles of 35°, 180° and 325°. A range shifter of 5.7 cm water-equivalent thickness was used for anterior-oblique fields. Typical proximal, distal and lateral target margins were 0.5 cm, 0.5 cm and 1 cm, respectively.

#### 5.2.3. Center-C

IMPT plans utilized a SIB, delivering 70 Gy, 60/58 Gy and 54 Gy to PTV_B_, PTV_E1_ and PTV_E2_ in 35 fractions, respectively. PTV D95% aim was ≥95% of the prescribed dose. IMPT plans were created using Varian Eclipse NUPO and PCS v13.7.15 with a 2.5 mm dose calculation grid. Plans typically utilized a four-field MFO with varying gantry angles to cover both cranial and caudal portions of a patient. Beam-arrangements were cross shaped in cranial portions to avoid the shoulders. A range shifter of 5 cm water equivalent thickness was used for all fields. Each field included proximal, distal and lateral target margins of 0.8 cm, 0.8 cm and 1.7 cm, respectively. 

### 5.3. Evaluation Patients

Each external center provided 7 HNC, non-robustly optimized, coplanar IMPT benchmark plans. Center-A provided clinical plans while center-B and -C created plans solely for the purpose of this study. Benchmark plans were deemed clinically acceptable by each of the respective external centers. All centers used their own clinical protocol and beam arrangement. For each patient, and without reference to the benchmark plans, RapidPlanPT was used to create non-robust KBPs using NUPO v15.5.99 (facilitates use of line objectives) and PCS v15.0.17 using a standard Y-shaped beam arrangement and the same target margins as used in the model-plans. Dose values of PTV_E1_/PTV_E2_ optimization objectives were assigned manually, prior to optimization, as the prescription dose varied per center. Optimization objective priorities mirrored those of model library plans. A subsequent optimization or “continue optimization” was performed if (i) PTV homogeneity was not acceptable (priorities increased/objectives added for PTVs) or (ii) KBPs did not meet criteria in Table 2 for OARs not included in the model (objectives added for any non-compliant OAR outside of the modeled structures). External centers could utilize a number of volume constraints for OARs not included in Table 2, such as the brachial plexus. Where necessary, additional manual constraints were used in the KBP planning process to address this.

### 5.4. Study Endpoints

KBPs were normalized such that PTV_B_ D95% ≥ 100%, V95% ≥ 99% and D95% ≥ 95% for center-A, -B and -C, respectively. Since external centers had differing PTV and OAR planning aims (Table 2), comparisons between benchmark plans and KBPs were performed altogether and per-center, on the basis of (1) target coverage and homogeneity (PTV D95%, PTV V95% and PTV homogeneity index (HI), where HI = 100% × (D2% − D98%)/D50%) (2) mean dose to OARs. For combined data and data per center, paired 2-sided Student t-tests and Wilcoxon signed rank tests were used to determine whether differences between benchmark plans and KBPs were significant (*p* < 0.05), respectively. The time required to create KBPs was also measured.

### 5.5. Ethical Statement

All patients signed a consent form, where they agreed to the use of their data for scientific purposes at PSI. The institutional review board (IRB) of the University of Cincinnati Medical Center determined the research proposal does not meet the regulatory criteria for research involving human subjects and thus ongoing IRB oversight was not required. The retrospective planning study was approved at the University of Pennsylvania under IRB # 830036 (1 June 2018).

## Figures and Tables

**Table 1:** Dosimetry averaged for the 7 benchmark plans and KBPs from each of the three external centers. Wilcoxon signed rank tests were used to identify significant (*p* < 0.05) differences between benchmark plans and KBPs, with * indicative of such differences.

	Center A		Center B		Center C	
PTVs	Benchmark	KBP	Benchmark	KBP	Benchmark	KBP
PTVB D95 (%)	100 ± 0	99.8 ± 0.6	97.5 ± 1.2	97.6 ± 0.4	97.8 ± 0.5	98 ± 0.3
	(100–100)	(98.36–100)	(95–98.4)	(97.3–98.3)	(97.2–98.6)	(97.6–98.6)
PTVB V95 (%)	100 ± 0	99.8 ± 0.5	99.5 ± 0.4	99.1 ± 0.2 *	99.7 ± 0.3	99.6 ± 0.4
	(100–100)	(98.7–100)	(99–100)	(99–99.5)	(99.2–100)	(99–100)
PTVB MaxDose (Gy)	75.4 ± 0.9	75.9 ± 0.9	74.7 ± 1.7	77.7 ± 1.4 *	74.5 ± 1.2	75.8 ± 1.8
	(74.2–77)	(75.2–77.5)	(72.2–77.6)	(75.1–79.8)	(72.7–76.2)	(73.9–78.9)
PTVB MinDose (Gy)	66 ± 2.3	64.9 ± 2.8 *	62.2 ± 2	56.5 ± 4 *	62.1 ± 2.3	60.5 ± 4.8
	(62–68.7)	(60–67.7)	(59.6–65.2)	(52–62.9)	(58.9–64.7)	(53–65.8)
HIB (%)	6.2 ± 1	7.6 ± 1.5	5.3 ± 1.2	7 ± 0.7 *	5.7 ± 1.3	5.8 ± 1.3
	(4.9–7.5)	(6.2–10.3)	(3.9–7.2)	(6–8.1)	(3.6–7.2)	(4.9–8.4)
PTVE1 D95 (%)	100.6 ± 0.9	101.3 ± 1	99.5 ± 0	98.1 ± 0	101.2 ± 2.2	101.1 ± 1.8
	(99.4–101.8)	(100–102.5)	(99.5–99.5)	(98.1–98.1)	(99.1–104.8)	(98.7–103.4)
PTVE1 V95 (%)	99.8 ± 0.2	99.6 ± 0.3	100 ± 0	99.3 ± 0	99.6 ± 0.3	99.6 ± 0.3
	(99.5–99.9)	(99.2–99.9)	(100–100)	(99.3–99.3)	(99.2–100)	(99.2–100)
HIE1 (%)	20.7 ± 2.6	21.3 ± 2.4	10.2 ± 0	10.7 ± 0	18 ± 2.7	17.4 ± 2
	(15.4–22.8)	(16.1–23.1)	(10.2–10.2)	(10.7–10.7)	(13.7–20.9)	(15.4–20)
PTVE2 D95 (%)	99.9 ± 1.5	100.7 ± 1.7	98.8 ± 0.4	98 ± 0.7 *	98.7 ± 1.1	98.5 ± 0.8
	(97.2–101.9)	(98.3–103)	(98.1–99.3)	(97.1–98.9)	(97.4–100.4)	(97.6–99.9)
PTVE2 V95 (%)	99.4 ± 1	99.8 ± 0.1	99.7 ± 0.2	98.9 ± 0.6 *	99.2 ± 0.7	99.2 ± 0.6
	(97.1–100)	(99.7–99.9)	(99.3–99.9)	(97.8–99.5)	(98.3–99.9)	(98.4–99.9)
HIE2 (%)	26.4 ± 3.6	24.3 ± 5.5	22.7 ± 2	22.5 ± 1.7	25.6 ± 8.8	24.1 ± 8.4
	(20.3–29.3)	(15.8–31.2)	(20–25.1)	(19.4–24.7)	(7.7–32.8)	(13–32.8)
**OAR Mean Doses (Gy)**						
Contra. Parotid	16.5 ± 7.3	17.7 ± 8.7	20.5 ± 14.4	12.3 ± 8.7 *	14.07 ± 7.55	13.2 ± 8.7
	(6.4–25.7)	(6.3–29.5)	(3.5–48.4)	(2–26.8)	(4–23.6)	(4.3–27.8)
Ipsi. Parotid	25.1 ± 3.2	28 ± 3.3 *	31.2 ± 11.6	20.8 ± 7.6 *	28.9 ± 23	27.3 ± 17.5
	(21.7–31.5)	(24–33)	(13.1–49.7)	(7.4–30.8)	(5.6–61.1)	(7–51.8)
Contra. Sub	45.1 ± 14.9	42.5 ± 15.6	51.5 ± 11.8	35.2 ± 24.8 *	53.6 ± 7.3	45.5 ± 12.1 *
	(28–64.9)	(24–63.3)	(39–68.4)	(11.7–66.5)	(43.1–64.7)	(32.3–63)
Ipsi. Sub	63.1 ± 8.1	60 ± 7.81	67.9 ± 2.7	66.9 ± 4.2	64.2 ± 5.9	63.5 ± 6.4
	(47.3–68.9)	(44.4–65)	(65.5–70.5)	(61.1–70.1)	(54.4–69.9)	(52.6–68.8)
Oral Cavity	10.5 ± 5.5	13.5 ± 7.7 *	23.2 ± 18.4	16.5 ± 20.3 *	28.4 ± 12.2	27.9 ± 15.5
	(2.2–16.2)	(3.2–22.4)	(10.3–63.8)	(3.1–61.2)	(8.5–43.7)	(3.7–47.7)
Constrictors	40.6 ± 5.4	38.5 ± 6.7	42.2 ± 15.7	30.4 ± 11	50.1 ± 8.5	39.5 ± 16.7 *
	(32.5–48.5)	(31–47.3)	(18.7–51.4)	(15.2–41.4)	(40.8–63)	(18–59.8)
Esophagus	17 ± 9.7	12.8 ± 9.1 *	16.7 ± 8.3	7.1 ± 4.8 *	12.4 ± 13.4	7.2 ± 6.3
	(8.3–35.8)	(5.9–30)	(1.2–25.4)	(0.4–15.8)	(1.5–31)	(0.4–13.2)
Larynx	28.5 ± 6.9	25.2 ± 7 *	22.2 ± 10.8	9.6 ± 3.4 *		
	(21.6–40.3)	(18.7–39.6)	(5.2–36)	(4–14.3)		
Glottis					53.7 ± 16.3	50.9 ± 18.3
					(29.9–68.5)	(21.7–68.1)
**OAR Max Doses (Gy)**						
Spinal Cord	39 ± 5.2	38.8 ± 3.4	45 ± 3.2	34.4 ± 4.5 *	40.9 ± 3.8	36.7 ± 8.7
	(28.5–42)	(34.7–43.1)	(41.9–50.7)	(27.8–38.7)	(37.5–49)	(19.1–47.5)
Brainstem	43.4 ± 7.3	42.5 ± 5.1	34.2 ± 12.6	27.3 ± 6.7	34.5 ± 15.3	22 ± 18.8 *
	(34.4–53)	(33.7–48.3)	(15.8–54.2)	(18.3–36.7)	(14.8–52.4)	(0–41.5)

**Table 2:** General planning-aims for the three external centers. *: Center A constraints for organs-at-risk (OARs) are on OARminusPTV delineations. We report dosimetric results based on entire OAR structures.

PTVs	Center A *	Center B	Center C
D95%	≥100%		≥95%
V95%		≥99%	
Dmax	≤110%	≤115%	
D99%	≥93%		≥90%
D2%			≤107%
D5%			≤105%
**Max Dose (Gy)**			
Spinal Cord	≤45	≤45	≤41
Spinal Cord PRV	≤50		
Brainstem	≤54	≤54	≤62
Brain		ALARA	≤65
Optic Nerve	≤54	≤54	≤60
Chiasm	≤54	≤54	≤60
Esophagus			≤71.4
Larynx			≤71.4
TMJoint			≤70
Trachea			≤71.4
Eye			≤50
Mandible-PTV	≤70		
**Mean Dose (Gy)**			
Parotid	<26	<26	<24
Submandibular	<30	<26	
Oral Cavity	<20	<30	<45 (<35 if distal to target)
Pharynx	<50		<45
Larynx	<20	<30	
Esophagus	<20	<30	<40 if not in PTV
Middle Ears	<30	<30	
Eye	<35	ALARA	<35
Glottis			<45 (<20 if distal to target)
Trachea			<40

## Supplementary Materials

The following are available online at http://www.mdpi.com/2072-6694/10/11/420/s1, Figure S1: Dose-volume histograms (DVHs, solid lines) and respective DVH-predictions (shaded regions) of patient 1 for (LEFT) the non-robust IMPT KBP when using the CTV as a target volume and (RIGHT) the robustly-optimized (4 mm, 3%) IMPT KBP when using the CTV as a target volume, Figure S2: DVH-predictions (shaded regions) and respective DVHs (solid lines) for the robustly optimized (4 mm, 3%) IMPT KBP of patient 1 when using the CTV (▲) and CTV + 2 mm (■) as model targets. Both plans were optimized using the original CTV (Same patient as in Figure S1), Table S1: Robustness evaluation (isocenter shift 4 mm, calibration curve error 3%) and nominal dosimetry for robust IMPT KBPs created using the CTV, CTV + 1 mm (CTV + 1), CTV + 2 mm (CTV + 2), CTV + 3mm (CTV + 3) and CTV + 4 mm (CTV + 4) to generate OAR DVH-predictions, averaged over three validation patients, Table S2: Robustness evaluation (isocenter shift 3 mm, calibration curve error 3.5%) and nominal dosimetry for robust clinical plans and KBPs for three patients from center-A.
