# Supplementary material for: Automated Knowledge-Based Intensity-Modulated Proton Planning: An International Multicenter Benchmarking Study
Source: Cancers (Basel). 2018 Nov 2;10(11):420. doi: 10.3390/cancers10110420 (PMC6266684; doi:10.3390/cancers10110420)
Supplement: Supplementary file 1 [file cancers-10-00420-s001.pdf]

Supplementary Materials: Automated Knowledge-Based Intensity-Modulated Proton Planning: An International Multicenter Benchmarking Study

Alexander R Delaney, Lei Dong, Anthony Mascia, Wei Zou, Yongbin Zhang, Lingshu Yin, Sara Rosas, Jan Hrbacek, Antony J Lomax, Ben J. Slotman, Max Dahele and Wilko F.A.R. Verbakel

1. Using an IMPT model consisting of non-robust plans to generate robust IMPT treatment plans

RapidPlan uses geometric characteristics from plans in a model library, including the target volume, overlap volume of an organ-at-risk (OAR) with the target volume, and the distance from an OAR to a target volume, to predict the dosimetry for a prospective patient. Robust proton optimization is typically based on the clinical target volume (CTV), rather than the planning target volume (PTV). While many proton centers may have an extensive library of non-robustly optimized treatment plans, their library of robustly-optimized plans may be quite modest due to the relatively recent progressing transition to robust optimization. Since the proton model discussed in this study comprises non-robust intensity-modulated proton therapy (IMPT) treatment plans optimized on the PTV, the question is if such a model can also be used to generate predictions for robust IMPT optimization, without the need to generate a new library of robustly optimized IMPT plans.

To investigate this, we first tested if the non-robust model could be used to create non-robust IMPT treatment plans where the plan is optimized on the CTV only. Applying this 50-patient IMPT model on three validation patients and using the CTV to generate predictions, led to acceptable CTV coverage and OAR dose-volume histogram (DVH)-predictions representative of what was achieved after optimization (Figure S1, left). The next step was to try to use the same approach to generate OAR DVH-predictions for robust optimization, under uncertainty conditions of 4 mm and 3 %. When using the CTV as the target, the OAR DVH predictions were too low in comparison with the final achieved DVHs (Figure S1, right).

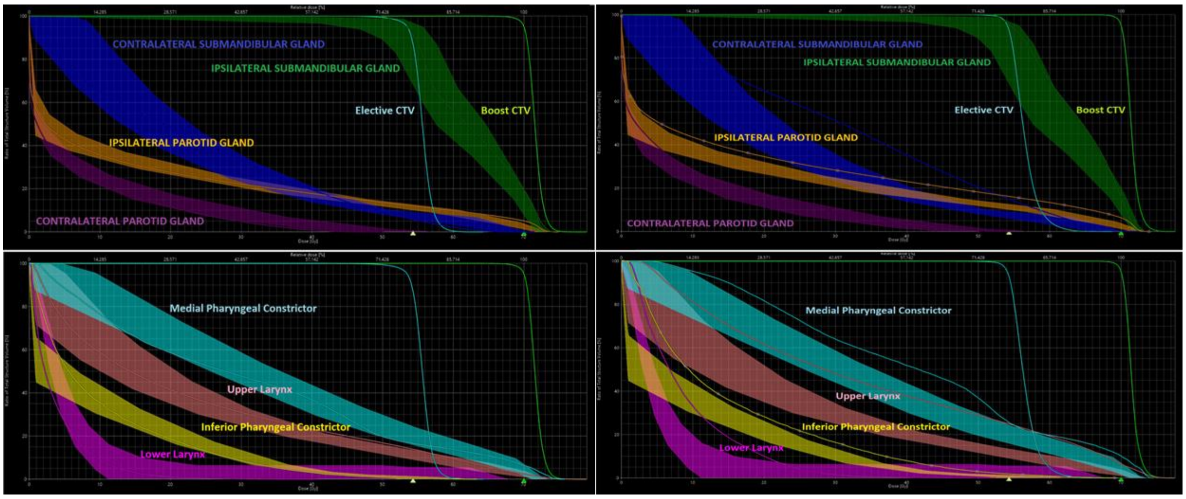

Figure S1. Dose-volume histograms (DVHs, solid lines) and respective DVH-predictions (shaded regions) of patient 1 for **(LEFT)** the non-robust IMPT KBP when using the CTV as a target volume and **(RIGHT)** the robustly-optimized (4mm, 3%) IMPT KBP when using the CTV as a target volume.

We postulated that using a larger target structure would shift predictions upwards and increase prediction accuracy, and therefore optimization-objective placement. To test this, CTV expansions of 1 mm, 2 mm, 3 mm and 4 mm were used to as target structures when generating DVH-predictions

for OARs and results were compared to that of using the original CTV, for three patients. While predictions were generated using these expanded CTV structures, optimization-objectives for the target were applied to the original CTV, under the uncertainty conditions of 4 mm and 3%. It was found that assigning the original CTV or an expansion of 1 mm as the target structure typically led to an overestimation of OAR sparing while using an expansion of 4 mm led to an underestimation. 2 mm seemed to provide the best balance between the DVH-prediction accuracy and CTV coverage under uncertainties (4 mm, 3 %) in this patient sample (Figure S2 and Table S1).

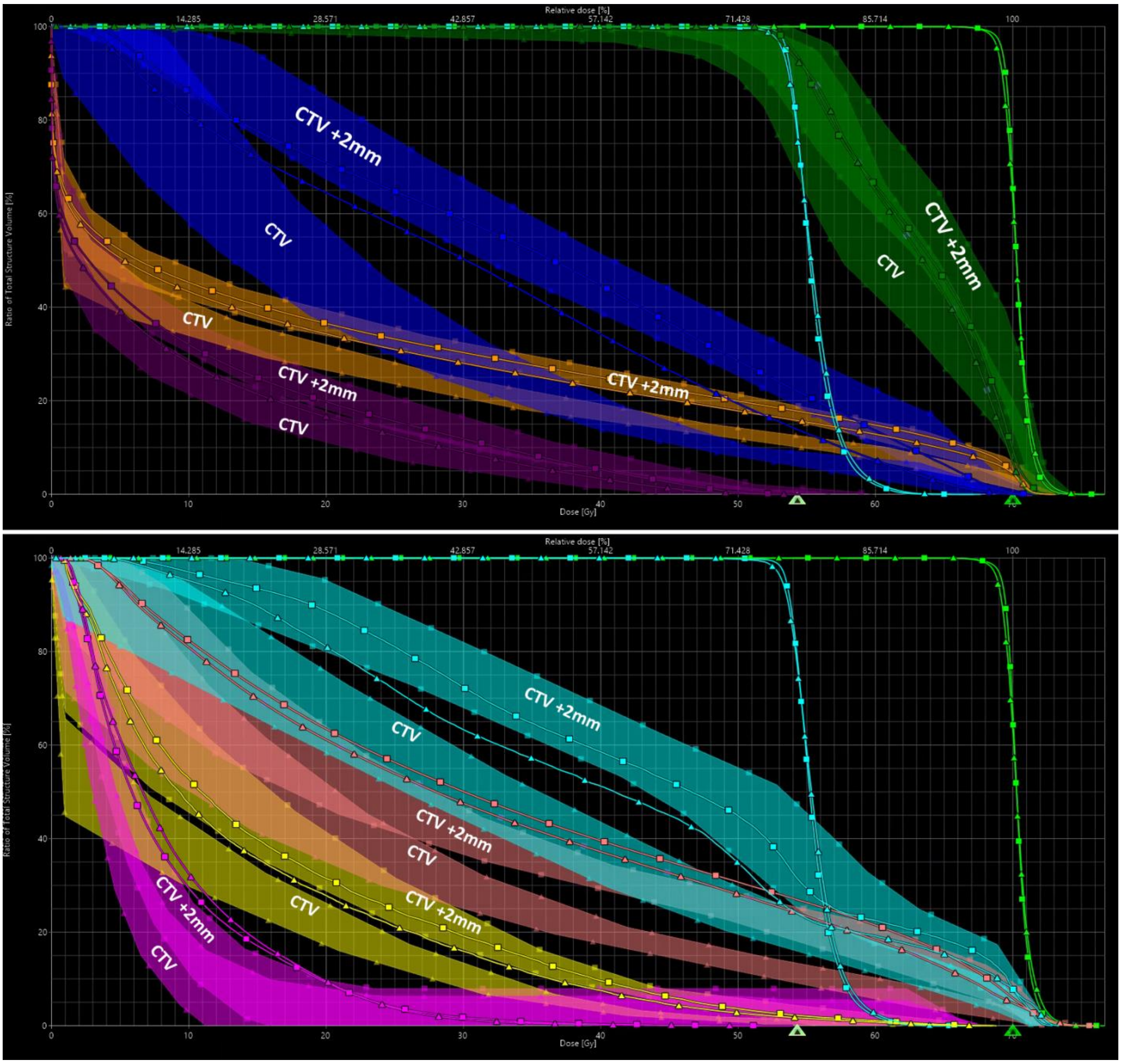

Figure S2. DVH-predictions (shaded regions) and respective DVHs (solid lines) for the robustly optimized (4 mm, 3%) IMPT KBP of patient 1 when using the CTV (▲) and CTV +2 mm (■) as model targets. Both plans were optimized using the original CTV (Same patient as in Figure S1).

Table S1. Robustness evaluation (isocenter shift 4 mm, calibration curve error 3%) and nominal dosimetry for robust IMPT KBPs created using the CTV, CTV + 1 mm (CTV + 1), CTV + 2mm (CTV + 2), CTV + 3 mm (CTV + 3) and CTV + 4 mm (CTV + 4) to generate OAR DVH-predictions, averaged over three validation patients.

	KBP CTV	KBP CTV + 1	KBP CTV + 2	KBP CTV + 3	KBP CTV + 4
**V95 of worst DVH (%)**					
CTVB	98.01	98.58	98.83	98.96	99.17
CTVE	96.27	97.24	97.66	98.04	98.39
**Nominal Data**					
CTVB V95 (%)	99.99	99.99	100.00	100.00	99.99
HIB (%)	6.59	6.04	5.67	5.50	5.72
CTVE V95 (%)	99.66	99.79	99.85	99.89	99.93
HIE (%)	13.92	13.17	13.10	13.02	13.04
**OAR Mean Doses (Gy)**					
Contra. Parotid	10.05	10.67	11.23	11.91	13.01
Ipsi. Parotid	23.88	24.77	25.23	25.79	26.55
Contra. Sub	37.47	38.56	39.50	40.68	42.43
Oral Cavity	8.14	7.91	7.91	7.84	8.35
Cricoph	14.42	15.37	15.91	16.67	20.11
PCM Inferior	22.21	24.34	25.30	26.80	28.97
PCM Medial	43.94	46.37	47.70	49.04	50.39
PCM Superior	33.44	34.44	35.03	35.67	37.32
Lower Larynx	13.59	14.42	15.02	15.58	17.29
Upper Larynx	49.32	50.94	51.45	52.15	52.89
UES	11.43	12.69	13.49	14.30	16.00
**OAR Max Doses (Gy)**					
Spinal Cord	32.64	31.98	32.15	32.44	30.21
Brainstem	13.98	13.45	13.38	12.65	12.38

2. Creating robust KBPs for an external center

To further validate the above mentioned methodology of using a model based on non-robustly optimized plans for robust optimization, Center-A provided robustly optimized clinical treatment plans for three new patients for benchmarking purposes. Robust KBPs were created for each of these three patients using the standard field set-up, target margins and optimization/dose calculation algorithm outlined in the main manuscript in conjunction with the use of CTV + 2 mm expansion as the target for generating the DVH predictions, as explained above. A "continue optimization" was performed in all three cases to improve CTV homogeneity. This was done by increasing the priorities of CTV objectives and converting any high-dose isodose lines in the CTV to contours and applying an upper dose-volume constraint. Clinical treatment plans and KBPs were compared on the basis of target coverage and homogeneity and mean dose to OARs. Since Center-A evaluates the robustness of their clinical treatment plans under 3 mm isocenter shifts and 3.5 % calibration curve errors, with acceptable criteria being that the second worst error scenario delivers 95% of the prescribed dose to at least 95% of the CTV volume, both sets of plans were evaluated for robustness using these criteria.

Table S2 shows results for the robustness of both clinical plans and KBPs as well as target quality indices and OAR dosimetry in the nominal scenario. All clinical plans and KBPs met robustness criteria (V95% of 2nd worst DVH >95% of CTV prescription dose). And while the KBP for patient 2 met robustness criteria and nominal CTV doses provided similar coverage, CTV homogeneity was inferior to that of the clinical plan. Differences in mean dose between clinical plans and KBPs were <4 Gy in 15/21 OARs. In 3/6 of the remaining OARs there were improvements in mean dose in the KBPs. However, the remainder included a 7 Gy and 9 Gy increase in oral cavity and larynx mean dose respectively, in the KBP for patient 2. Lastly, larynx mean dose increased by 9 Gy in the KBP for patient 3. The increase in oral cavity mean dose can be attributed to the use of two anterior oblique fields in the KBP, compared with the clinical plan, in which anterior fields are absent in the cranial portion. Likewise, the increases in larynx mean dose were contributed to by the differing beam arrangements between plans. In all 3 patients, spinal cord and brainstem maximum dose was lower (16 Gy and 24 Gy on average, respectively) in the KBPs.

These preliminary tests suggest it is feasible to use RapidPlan for robust optimization. Furthermore, in the case that there exists no suitably large library of robustly optimized plans to generate a model, it appears possible to use a model comprising non-robust plans.

Table S2. Robustness evaluation (isocenter shift 3 mm, calibration curve error 3.5%) and nominal dosimetry for robust clinical plans and KBPs for three

	**Patient 1**		**Patient 2**		**Patient 3**	
	**Robust Clinical Plan**	**Robust KBP**	**Robust Clinical Plan**	**Robust KBP**	**Robust Clinical Plan**	**Robust KBP**
**V95 of 2nd Worst DVH (%)**						
CTVB	97.1	98.36	98.2	96.9	96.58	98.33
CTVE1	97.6	97.08			99.2	99.57
CTVE2	96.1	96.05	98.1	95.11	97.4	98.8
**Nominal Data**						
CTVB D95 (%)	100	100	100	100	100	100
CTVB V95 (%)	99.04	99.97	99.92	99.9	100	100
CTVB MaxDose (Gy)	69.2	69.61	65.46	68.25	74.76	77.52
CTVB MinDose (Gy)	46.08	55.37	52.92	50.99	66.43	65.76
HIB (%)	8.9	5.8	5.36	10.48	5.61	6.85
CTVE1 D95 (%)	99.65	98.98			101.6	102.62
CTVE1 V95 (%)	99.77	99.73			99.98	100
HIE1 (%)	8.77	9.81			19.57	19.79
CTVE2 D95 (%)	99.34	99.79	100.44	98.81	101.38	101.68
CTVE2 V95 (%)	98.84	99.77	99.98	99.65	99.81	99.98
HIE2 (%)	14.24	12.07	10.61	14.61	18.42	17.35
**OAR Mean Doses (Gy)**						
Contra. Parotid	18.55	8.23	4.71	3.18	13.16	12.08
Ipsi. Parotid	26.14	27.81	22.43	24.89	25.34	26.42
Contra. Sub	55.7	54.94	18.4	19.94	29.32	32.88
Oral Cavity	9.52	12.92	1.47	8.73	8.41	11.52
Constrictors	35.03	25.66	18.97	20.9	27.64	30.95
Esophagus	7.67	8.02	11.53	4.62	16.64	16.4
Larynx	24.88	27.98	12.71	21.32	21.36	30.31
**OAR Max Doses (Gy)**						
Spinal Cord	44.89	25.18	38.7	18.38	4.92	34.74
Brainstem	46.84	22.74	34.92	10.46	12.98	23.1
